# Exploring PIM1 Kinase as a Therapeutic Target: Mechanisms and Strategies in Cancer Treatment

**DOI:** 10.3390/ijms27146303

**Published:** 2026-07-15

**Authors:** Tingyu Zeng, Huayong Liu, Zhipan Li, Tiange Liu, Kaifeng Zhang, Shuping Wang

**Affiliations:** Key Laboratory of Preclinical Study for New Drugs of Gansu Province, Institute of Biochemistry and Molecular Biology, School of Basic Medical Sciences, Lanzhou University, Lanzhou 730000, China; tingyu_zeng@126.com (T.Z.); 17806087313@163.com (H.L.); lzhipan2023@lzu.edu.cn (Z.L.); liutg2023@lzu.edu.cn (T.L.); zhangkf2023@lzu.edu.cn (K.Z.)

**Keywords:** stemness, therapeutic resistance, PIM1, targeted therapy, immune response

## Abstract

Cancer remains a major global health challenge and is the second leading cause of death worldwide. Targeted therapy has emerged as one of the most promising strategies for cancer treatment. However, current targeted therapies highlight the urgent need for novel therapeutic targets and strategies. The provirus integration site for Moloney murine leukemia virus 1 (PIM1) kinase has been identified as a key factor in tumor progression and poor prognosis. This review systematically summarizes and analyzes the diverse mechanisms of PIM1 in promoting tumor progression, including cell programmed death, cell cycle progression, DNA damage response, metastasis, cell stemness, metabolic reprogramming, tumor angiogenesis, anti-cancer immune response and therapeutic resistance, and comprehensively evaluates its potential as a therapeutic target. Moreover, PIM1 contributes to the development of resistance to various anticancer therapies. Based on the advances and limitations in PIM1-targeted cancer therapy, we propose that future research should focus on combination strategies involving PIM1 inhibitors and agents targeting parallel or upstream/downstream pathways regulated by PIM1. Our review highlights the therapeutic value and potential of PIM1 in cancer treatment, providing new insights and theoretical bases for the development of novel anti-tumor strategies targeting PIM1.

## 1. Introduction

Cancer remains the second leading cause of death worldwide, with tumors capable of originating from nearly any organ or tissue in the body. This occurs when abnormal cells grow uncontrollably, surpassing their normal boundaries, invading adjacent tissues, and/or spreading to distant organs, a process known as metastasis, which is the primary cause of cancer-related mortality [1]. Traditional cancer treatment modalities include surgery, chemotherapy, and radiotherapy [2]. While surgery and radiotherapy are effective local treatments for primary tumors, they are limited in their ability to address metastatic or micrometastatic lesions [3]. Moreover, radiotherapy can lead to severe complications such as radiation-induced pneumonitis, which may be life-threatening [4]. Chemotherapy, as a systemic approach, can target subclinical lesions and prevent recurrence and metastasis, but its non-selective cytotoxicity also damages normal cells and tissues [5]. With the advent of high-throughput omics technologies, targeted therapies and immunotherapies have brought new hope to cancer treatment. Targeted therapies offer precision by specifically acting on molecular targets unique to cancer cells, thereby reducing collateral damage to normal tissues and minimizing the side effects associated with conventional chemotherapy [6]. Immunotherapies harness the immune system of patients to recognize and eliminate tumor cells, often resulting in more durable responses [7]. However, both approaches face significant challenges. Targeted therapies are often limited by the emergence of drug resistance and a scarcity of actionable targets [6]. Immunotherapies are hampered by variable response rates and immune-related adverse events [8]. Thus, the identification of novel therapeutic targets and strategies remains an urgent need in the effective management of cancer.

Provirus integration site for Moloney murine leukemia virus 1 (PIM1) is a serine/threonine protein kinase and a member of the PIM kinase family, recognized for its pivotal role in oncogenic signaling [9]. PIM1 is overexpressed in a variety of tumors, which is primarily attributable to its transcriptional activation and post-translational modifications at the protein level. Transcriptional activation is mainly mediated by the janus kinase (JAK)/signal transducer and activator of transcription (STAT) and nuclear factor kappa-B (NF-κB) pathways [10,11]. In addition, SUMOylation of PIM1 [12] enhances its kinase activity, thereby sustaining the high expression of PIM1 in tumor cells. In multiple tumor types, by phosphorylating a diverse array of substrate proteins, PIM1 regulates cell cycle progression, promotes cell survival, and mediates cellular responses to external stimuli [13]. Beyond these fundamental functions, PIM1 is critically involved in metabolic reprogramming, immune regulation, and various forms of programmed cell death [14,15]. Recent research has highlighted the importance of PIM1 in the initiation and progression of multiple tumor types. As a proto-oncogene, elevated PIM1 expression is closely linked to enhanced tumor cell proliferation, invasion, metastasis, therapeutic resistance, stemness, and modulation of the tumor microenvironment (TME) [16,17,18,19,20,21]. In non-small cell lung cancer (NSCLC), PIM1 promotes cell proliferation, metastasis, and tumor growth by augmenting cellular mesenchymal-epithelial transition factor (c-MET) signaling [22]. In T-cell lymphoma, PIM1 confers resistance to apoptosis and doxorubicin by upregulating cellular myelocytomatosis oncogene (c-Myc) expression, thereby protecting tumor cells from chemotherapeutic stress [23]. Overexpression of PIM1 in triple-negative breast cancer (TNBC) is associated with increased tumor cell proliferation and anti-apoptotic capacity, while inhibition of PIM1 reduces cell viability and sensitizes cells to chemotherapy [24]. In prostate cancer, PIM1 collaborates with c-Myc to drive tumorigenesis and progression, and further enhances cytoskeletal dynamics through phosphorylation of Abl interactor 2 (ABI2), facilitating tumor cell invasion [18,25]. PIM1 also regulates glycolytic pathways to promote cell proliferation and tumor formation in ovarian and colorectal cancers (CRC) [26,27]. In glioblastoma, upregulation of PIM1 is closely correlated with tumor cell survival and proliferation [28]. Collectively, these findings highlight the multifaceted role of PIM1 in tumorigenesis, progression, and therapeutic resistance. Targeting PIM1 kinase activity represents a promising strategy for cancer treatment, as its inhibition can effectively suppress tumor cell proliferation and survival, enhance chemosensitivity, and potentially improve patient outcomes.

The development of PIM1 inhibitors is currently transitioning from preclinical research to early clinical evaluation, with the primary goal of exploiting the dual role of PIM1 kinase in tumor proliferation and immune regulation to develop novel anticancer agents. Several PIM1-targeted inhibitors have been developed, including interleukin-1 receptor-associated kinase 4 (IRAK4)/PIM1 inhibitors that block the toll-like receptor (TLR)/myeloid differentiation primary response 88 (MYD88)-mediated NF-κB pathway, showing promise in the treatment of rheumatoid arthritis and lymphoid malignancies [29]. Novel dual inhibitors targeting both PIM1 and fibroblast growth factor receptor 1 (FGFR1) kinases have demonstrated efficacy in suppressing CRC growth [30]. Given the strong association between PIM1 and malignant tumor progression, this review will summarize and evaluate the molecular events regulated by PIM1, assess its potential as a therapeutic target in cancer, and provide perspectives and recommendations for the development of PIM1-targeted therapeutic strategies and drug discovery.

## 2. Structural and Functional Characteristics of PIM1 Kinase

**Figure 1:**
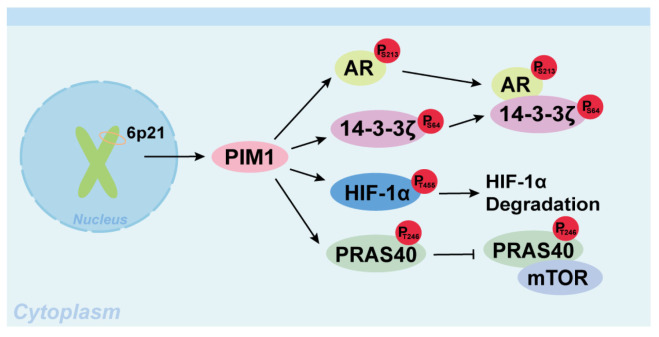
Structural and functional characteristics of PIM1 kinase. The PIM1 gene is located in the human chromosome region 6p21. PIM1 kinase phosphorylates AR at Ser213, 14-3-3ζ at Ser64, HIF-1α at Thr455, and PRAS40 at Thr246, stabilizing HIF-1α, activating mTOR, and enhancing AR signaling. These events promote proliferation, survival, migration, and metabolic adaptation of cancer cells.

PIM1 kinase is a serine/threonine kinase belonging to the PIM kinase family, which also includes PIM2 and PIM3 [31]. All three kinases are members of the Ca^2+^/calmodulin-dependent protein kinase family and share conserved serine/threonine kinase activity despite the existence of multiple isoforms with varying molecular weights [10]. The PIM1 gene is located in the human chromosome region 6p21 [32]. PIM1 is significantly overexpressed in hematological malignancies and various solid tumors, including prostate cancer, breast cancer, pancreatic cancer, osteosarcoma, and neuroblastoma [33]. PIM1 primarily exerts its oncogenic effects by phosphorylating a series of downstream substrates involved in tumor cell invasion and migration, cell cycle progression, and apoptosis. Similarly, PIM2 is located on the X chromosome and is widely expressed in hematological malignancies and various solid tumors, including prostate, breast, liver, and lung cancers [34,35]. PIM2 primarily exerts its oncogenic function by transcriptionally activating genes involved in cell survival and cell cycle progression. PIM3 is located on chromosome 22q13 and shares 71% amino acid identity with PIM1 [10]. PIM3 is predominantly overexpressed in liver, pancreatic, colon, gastric, prostate, and breast cancers. PIM3 plays multiple roles in promoting tumor proliferation, survival, invasion, metastasis, resistance to radiotherapy and chemotherapy, and the formation of an immunosuppressive microenvironment [36]. The three PIM family members possess highly similar kinase domains [37]. Although PIM2 shares only 55% amino acid identity with PIM1, it is regarded as a compensatory protein for PIM1 owing to their highly similar kinase domains [38]. The third member of the family, PIM3, has been shown to catalyze histone phosphorylation and autophosphorylation [39]. All three PIM proteins phosphorylate serine and threonine residues and activate similar cellular pathways [40]. This high degree of structural similarity has led researchers to propose that there is considerable functional redundancy among the PIM kinase family members. When PIM1 function is impaired or its expression is lost, other members can partially compensate for its function and activate similar signaling pathways, thereby maintaining essential cellular physiological activities. PIM1 depletion may lead to a compensatory response, involving the activation of PIM2 and PIM3. PIM1 contains a characteristic kinase domain, an adenosine triphosphate (ATP)-binding pocket, and an active site. In both mice and humans, alternative upstream CUG start codons generate two PIM1 protein isoforms, PIM-1L (44 kDa) and PIM-1S (34 kDa), both of which retain the kinase domain [41]. PIM-1S localizes to both the cytoplasm and the nucleus, whereas PIM-1L is localized to the plasma membrane [10]. Notably, PIM-1L possesses an additional N-terminal proline-rich proline-X-X-proline (PXXP) motif, enabling broader protein–protein interactions compared to PIM-1S [42]. Crystallographic studies reveal that PIM1 adopts a classical bilobal kinase structure, with an N-terminal lobe primarily composed of β-sheets and a C-terminal lobe dominated by α-helices, separated by a gap containing conserved ATP-binding residues. The two lobes are connected by a unique hinge region featuring a tertiary amine-containing proline residue, a distinctive hallmark of PIM kinases that may facilitate the development of highly selective PIM inhibitors [10]. PIM2 shares similar structural features with PIM1, and although the crystal structure of PIM3 remains unresolved, its high sequence homology with PIM1 suggests a comparable structural organization [36]. The primary post-translational modification mediated by PIM1 kinase is substrate phosphorylation, which plays a critical role in tumorigenesis and cancer progression [43]. For example, PIM1 phosphorylates the androgen receptor (AR) at serine 213 (pS213), and further phosphorylates the AR co-regulator 14-3-3ζ at S64 [44]. This phosphorylation enhances the interaction between AR and 14-3-3ζ, alters their chromatin occupancy, and upregulates genes involved in extracellular matrix organization, cell adhesion, and cytokine signaling, thereby increasing the migratory and invasive capacity of prostate cancer cells [43]. PIM1 also directly phosphorylates hypoxia-inducible factor 1-alpha (HIF-1α) to stabilize HIF-1α by blocking prolyl hydroxylase domain (PHD) binding, hydroxylation, ubiquitination, and subsequent proteasomal degradation, thus driving angiogenesis in solid tumors [44]. Additionally, PIM1 phosphorylates proline-rich Akt substrate of 40 kDa (PRAS40) at Thr246 independently of protein kinase B (AKT), reducing the association of PRAS40 with the mammalian target of rapamycin (mTOR) complex. Overexpression of PIM1 increases phosphorylation of mTOR substrates eIF4E-binding protein 1 (4E-BP1) and 70 kDa ribosomal protein S6 kinase (p70S6K) kinase, thereby enhancing mTOR pathway activity [45]. These findings highlight the multifaceted role of PIM1 in regulating key signaling pathways through substrate phosphorylation, contributing to cancer cell proliferation, survival, migration, and metabolic adaptation (Figure 1).

## 3. PIM1 Kinase Regulates Tumor Malignant Progression Through Multiple Pathways

PIM1 kinase plays a pivotal role in regulating the malignant progression of tumors through diverse mechanisms, including modulation of immune responses, therapeutic resistance, metabolic reprogramming, and cell cycle progression. By influencing these critical pathways, PIM1 contributes to tumor growth, survival, and adaptation in the TME. Consequently, targeting PIM1 offers a multifaceted approach to suppress tumor development and progression, highlighting its significance as a promising therapeutic target in cancer treatment.

### 3.1. The Role of PIM1 Kinase in Cell Programmed Death

**Figure 2:**
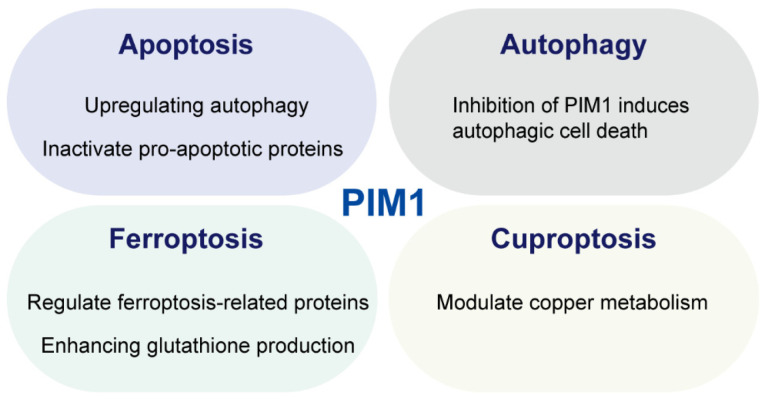
The Role of PIM1 Kinase in Cell Programmed Death. PIM1 kinase regulates cell programmed death through apoptosis, autophagy, ferroptosis and cuproptosis. PIM1 regulates apoptosis by blocking the function of pro-apoptotic proteins. PIM1 modulates autophagy via the REDD1/AMPK pathway. PIM1 suppresses ferroptosis by enhancing glutathione production. PIM1 modulates copper metabolism to regulate cuproptosis.

PIM1 is intricately involved in multiple forms of programmed cell death, including apoptosis, autophagy, ferroptosis, and cuproptosis, each of which plays a critical role in tumor progression. In apoptosis, a programmed cell death process triggered by intracellular and extracellular signals, PIM1 modulates the expression and activity of key apoptotic proteins, thereby influencing cell survival. For instance, PIM1 can phosphorylate and inactivate pro-apoptotic proteins such as bcl2-associated agonist of cell death (Bad), blocking apoptosis in gastric cancer cells [46]. Silencing PIM1 has been shown to inhibit proliferation and promote apoptosis in esophageal cancer cells [47]. Beyond direct regulation, PIM1 also interacts with various signaling molecules to regulate cell apoptosis [48]. In autophagy, a cellular degradation and recycling process essential for survival under stress conditions, PIM1 regulates the expression and activity of autophagy-related proteins and signaling pathways, facilitating autophagosome formation and degradation, and PIM1 overexpression inhibits apoptosis by upregulating autophagy. Notably, inhibition of PIM1 induces significant autophagic cell death, with evidence suggesting that PIM1 suppression activates regulated in development and DNA damage responses 1 (REDD1) and stimulates autophagy via the AMP-activated protein kinase (AMPK) cascade [49]. The role of PIM1 in ferroptosis, an iron-dependent form of cell death characterized by lipid peroxidation and membrane damage, is increasingly recognized. PIM1 influences ferroptosis by regulating the expression and activity of ferroptosis-related proteins and by enhancing glutathione production, thereby suppressing liver reprogramming-induced ferroptosis [50]. Additionally, PIM1 has been identified as a novel biomarker associated with ferroptosis and cuproptosis in abdominal aortic aneurysm (AAA), further underscoring its relevance in vascular pathology [51]. Cuproptosis, a newly described form of regulated cell death driven by copper ion accumulation and cellular damage [52]. This is another area where the regulatory mechanism of PIM1 is beginning to be clarified. Although the precise molecular details remain to be fully characterized, emerging evidence suggests that PIM1 may modulate copper metabolism and related signaling pathways, thereby influencing cellular susceptibility to cuproptosis [51,52]. Collectively, these findings demonstrate that PIM1 regulates a complex network of signaling pathways and molecular mechanisms governing various forms of programmed cell death (Figure 2). Elucidating the specific roles of PIM1 in apoptosis, autophagy, ferroptosis, and cuproptosis not only advances our understanding of tumor biology but also provides novel therapeutic opportunities for targeting PIM1 in cancer.

### 3.2. The Role of PIM1 Kinase in Cell Cycle Progression

Cell cycle refers to the complete process that a cell undergoes from the completion of one division to the end of the next, and is primarily divided into interphase and mitotic phase. Aberrant regulation of core cell cycle mechanisms is present in nearly all types of tumors and serves as a major driving force for tumorigenesis. PIM1 plays a pivotal role in modulating cell cycle progression and tumor cell proliferation. PIM1 accelerates cell cycle progression by phosphorylating key cell cycle regulatory proteins, such as p21Waf1 [42]. Pharmacological studies have demonstrated that the PIM1 inhibitor anwulignan can directly bind to PIM1, exerting significant antitumor effects on diffuse large B-cell lymphoma (DLBCL) both *in vitro* and *in vivo* by inducing apoptosis, cell cycle arrest, and autophagic cell death [53]. Furthermore, PIM1 deficiency leads to a significant reduction in B-cell receptor (BCR)-induced cell proliferation and cell cycle progression [14]. Collectively, these findings highlight the crucial role of PIM1 kinase in cell cycle and tumorigenesis, suggesting its potential as a promising therapeutic target in cancer treatment.

### 3.3. The Role of PIM1 Kinase in DNA Damage Response

DNA damage response (DDR) is an essential cellular mechanism that safeguards genomic integrity by detecting and repairing DNA lesions through a coordinated network of signaling pathways and repair processes. Among these, homologous recombination (HR) and non-homologous end joining (NHEJ) are pivotal for the repair of DNA double-strand breaks (DSBs). Recent research has highlighted PIM1 kinase as a critical regulator in the DDR, with emerging evidence demonstrating its involvement in multiple facets of DNA repair and cellular response to genotoxic stress [54]. PIM1 interacts with key proteins such as DNA-dependent protein kinase catalytic subunit (DNA-PKcs), a central component of the NHEJ pathway, potentially facilitating its recruitment and activation at sites of DNA damage to promote efficient DSB repair [55]. Additionally, PIM1 modulates chromatin structure through interactions with chromatin remodeling proteins like PDZ And LIM domain 1 (PDLIM1), thereby enhancing chromatin relaxation and accessibility for repair. Beyond direct protein interactions, PIM1 influences broader DDR signaling networks, including the regulation of ataxia telangiectasia mutated (ATM) kinase, a master orchestrator of the cellular response to DSBs, and its downstream effectors such as checkpoint kinase 2 (CHK2), mediator of DNA damage checkpoint protein (MDC1), and tumor protein p53 binding protein 1 (53BP1). This multifaceted involvement enables PIM1 to fine-tune cell fate decisions following DNA damage, balancing repair, cell cycle arrest, and apoptosis. The therapeutic potential of targeting PIM1 indicates that PIM1 inhibition sensitizes tumor cells to DNA-damaging agents, likely by impairing their DNA repair capacity [56]. Furthermore, combining PIM1 inhibitors with other DDR-targeting agents, such as poly ADP-ribose polymerase (PARP) inhibitors, may offer synergistic benefits, particularly in tumors with pre-existing DNA repair defects [57]. In summary, PIM1 serves as a crucial regulator of genomic stability through its direct and indirect roles in the DDR, positioning it as a promising target for cancer therapy.

### 3.4. The Role of PIM1 Kinase in Tumor Cell Senescence

**Figure 3:**
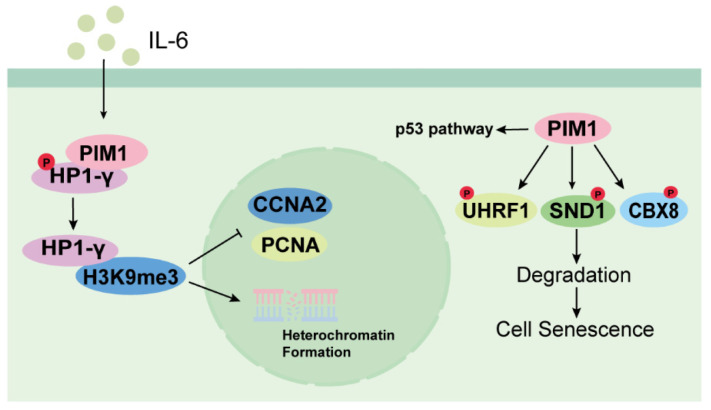
The Role of PIM1 Kinase in Tumor Cell Senescence. IL-6 promotes the protein interaction between PIM1 and HP1-γ, which recruits H3K9me3 to inhibit the expression of CCNA2 and PCNA and promote heterochromatin formation. PIM1 promotes the degradation of UHRF1, SND1, and CBX8 through phosphorylation, thereby enhancing cancer cell senescence.

Tumor cell senescence is a complex and multifaceted process that exerts both tumor-suppressive and tumor-promoting effects in cancer progression and therapy. Senescence is characterized by a stable cell cycle arrest, preventing the proliferation of damaged or oncogenically activated cells and thereby acting as a barrier to tumorigenesis. However, senescent cells can also contribute to tumor progression through the senescence-associated secretory phenotype (SASP), which involves the secretion of pro-inflammatory cytokines, growth factors, and proteases that promote tumor growth, metastasis, and therapeutic resistance [58]. The role of PIM1 kinase in tumor cell senescence has garnered significant attention due to its diverse functions in cellular proliferation, survival, differentiation, and apoptosis. Recent studies have elucidated the involvement of PIM1 in oncogene-induced senescence (OIS), where it acts as a tumor suppressor by halting the proliferation of cells with oncogenic alterations [59]. Mechanistically, PIM1 phosphorylates substrates such as staphylococcal nuclease domain-containing protein 1 (SND1), and its reduced expression leads to upregulation of SASP, highlighting the regulatory role of PIM1 in cellular senescence [59]. PIM1 also interacts with heterochromatin protein 1γ (HP1-γ), phosphorylating it to enhance binding to histone H3 lysine 9 trimethylation (H3K9me3) and promote heterochromatin formation, thereby suppressing proliferative gene expression and reinforcing senescence [60]. Furthermore, PIM1-mediated phosphorylation and degradation of ubiquitin-like with PHD and ring finger domains 1 (UHRF1) result in DNA hypomethylation and genomic instability, leading to increased multiple tumor cyclin-dependent kinase inhibitor 2A (p16) expression and senescence induction [61]. The regulation of PIM1 by external signals, such as interleukin-6 (IL-6) and oxidative stress, further integrates cytokine signaling and cellular stress responses into the senescence machinery [62,63]. Notably, PIM1 can also induce senescence by promoting the degradation of chromobox homolog 8 (CBX8), a transcriptional repressor of p16, and by modulating the p53 pathway [64]. In TNBC, targeting promyelocytic leukemia protein (PML) induces growth inhibition and senescence, with cyclin-dependent kinase inhibitor 1B (p27) identified as a key driver of PML-silencing-induced senescence [65]. Collectively, these findings highlight the regulatory network of PIM1 in regulating tumor cell senescence, positioning it as a promising therapeutic target for cancer (Figure 3).

### 3.5. The Role of PIM1 Kinase in Tumor Metastasis

PIM1 is closely associated with tumor cell metastasis, exerting its effects through multiple signaling pathways and molecular interactions in various cancers. Studies have demonstrated that PIM1 enhances the motility of prostate cancer cells and promotes migration and invasion in malignant mesothelioma, breast cancer, NSCLC, hepatocellular carcinoma (HCC), and other cancers [66,67]. In mesothelioma, PIM1 knockout leads to reduced cell proliferation, G1 cell cycle arrest, and diminished invasive and migratory capabilities [66]. PIM1 facilitates breast cancer cell migration via the Rho-associated coiled-coil-containing protein kinase 2 (ROCK2)/STAT3 pathway [68]. In NSCLC, PIM1 knockdown significantly impairs cancer cell migration and invasion [20]. In HCC, the interaction between PIM1 and RNA-binding motif protein, Y chromosome (RBMY) triggers RBMY phosphorylation, mitochondrial translocation, mitochondrial fission, and increased ATP and reactive oxygen species (ROS) synthesis, collectively driving tumor metastasis [19]. In prostate cancer, PIM1-mediated phosphorylation of N-myc downstream regulated 1 (NDRG1) reduces its stability, nuclear localization, and interaction with the AR, thereby enhancing cell migration and invasion [69]. Additionally, phosphorylation of nuclear factor of activated T cells 1 (NFATC1) by PIM1 stimulates its activity, further promoting metastatic potential [70]. PIM1 also regulates c-MET signaling in lung adenocarcinoma, facilitating cell proliferation, metastasis, and tumor growth. In clear cell renal cell carcinoma, PIM1 targets SMAD family member (SMADs) in the nucleus to mediate EMT and tumor progression [71]. Furthermore, in pancreatic ductal adenocarcinoma (PDAC), PIM1 regulates the Kirsten rat sarcoma viral oncogene homolog (K-Ras) signaling pathway, contributing to cell growth, invasion, and radioresistance [72]. In DLBCL, PIM1 collaborates with B-cell lymphoma 6 (BCL6) to facilitate lymphoma metastasis [73]. Collectively, these findings highlight the central role of PIM1 in regulating tumor cell migration, invasion and metastasis through multiple molecular mechanisms, making it a promising target for anti-metastatic cancer treatment.

### 3.6. The Role of PIM1 Kinase in Tumor Cell Stemness

**Figure 4:**
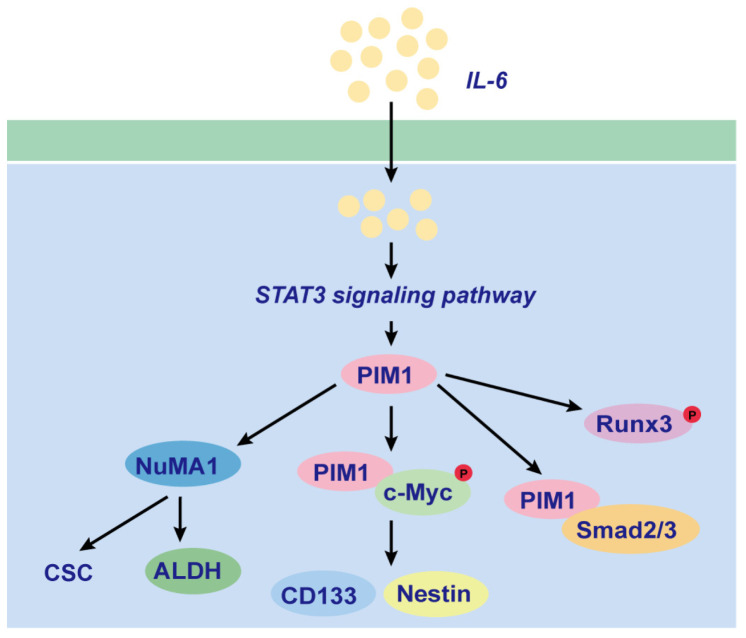
The Role of PIM1 Kinase in Tumor Cell Stemness. IL-6 promotes PIM1 expression through the STAT3 signaling pathway. PIM1 enhances cancer stem cell properties by interacting with c-Myc to upregulate CD133 and Nestin. PIM1 promotes RUNX3 cytoplasmic retention, interacts with Smad2/3 and maintains CSC through NuMA1.

Tumor cell stemness refers to the acquisition of stem cell-like properties by cancer cells, including self-renewal and multipotent differentiation capabilities, which play a pivotal role in tumor initiation, progression, and therapeutic resistance. This phenomenon is tightly regulated by a variety of signaling pathways and molecular mechanisms. Recent studies have demonstrated that PIM1 kinase is a key regulator of tumor cell stemness in multiple cancers. PIM1 promotes EMT and enhances stemness by activating transcription factors such as c-Myc, thereby increasing tumor invasiveness and therapeutic resistance [21]. In breast cancer, IL-6 upregulates PIM1 expression through the STAT3 pathway, which in turn activates c-Myc and induces EMT and stem cell marker expression, leading to increased invasion [21]. PIM1 also facilitates the phosphorylation and cytoplasmic localization of RUNX family transcription factor 3 (RUNX3), further augmenting stem cell-like traits in breast cancer cells [74]. Similarly, in clear cell renal cell carcinoma, PIM1 interacts with SMAD2/3 and c-Myc to drive the expression of EMT-related transcription factors and promote tumor progression [28]. In glioblastoma, PIM1 upregulates stem cell markers such as prominin-1 (CD133) and Nestin, and its inhibition significantly reduces glioblastoma stem cell survival and self-renewal [28]. The upstream regulation of nuclear mitotic apparatus protein 1 (NuMA1) by PIM1 is essential for maintaining breast cancer stem cell phenotypes, as NuMA1 deficiency leads to a reduction in acetaldehyde dehydrogenase (ALDH) and CD29^+^hiCD61 breast cancer stem cells (CSCs) [75]. PIM1 also plays a crucial role in prostate cancer cell stemness, potentially activating key signaling pathways through phosphorylation of targets such as breast cancer resistance protein (BCRP) and Myc [76]. Furthermore, combined inhibition of PIM1 and anti-apoptotic proteins enhances apoptotic sensitivity in tumor cells, suggesting the role of PIM1 in sustaining cancer cell survival [77]. Collectively, these findings highlight the strong association between PIM1 expression and tumor cell stemness, mediated through the regulation of critical signaling pathways and transcription factors (Figure 4).

### 3.7. The Role of PIM1 Kinase in Metabolic Reprogramming

**Figure 5:**
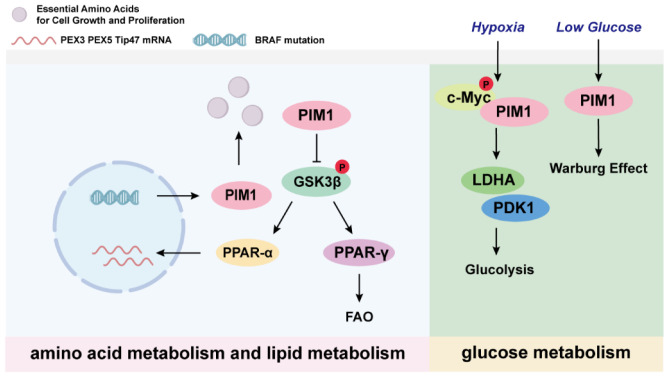
The Role of PIM1 Kinase in Metabolic Reprogramming. PIM1 promotes the Warburg effect and glycolysis under glucose deprivation or hypoxia. PIM1 drives lipid droplet accumulation via GSK3β/PPAR-α. PIM1 also induces PPAR-γ for fatty acid oxidation.

A hallmark of cancer cell metabolism is the ability to acquire essential nutrients from often nutrient-deprived environments and utilize these resources to sustain viability and generate new biomass. The metabolic reprogramming associated with cancer leads to profound alterations in both intracellular and extracellular metabolites, which in turn have significant impacts on gene expression, cellular differentiation, and the TME. PIM1 kinase plays a pivotal role in this metabolic adaptation in various malignancies. In glucose metabolism, PIM1 promotes tumor proliferation and resistance to glucose deprivation through the Warburg effect [27]. In CRC, glucose deprivation is one of the mechanisms driving increased PIM1 expression, and this upregulation compensates by enhancing the Warburg effect to ensure CRC growth under low-glucose conditions [27]. Recent studies have shown that PIM1 enhances the survival of PDAC cells under glucose deprivation by reducing ROS accumulation [15]. In HCC, hypoxia induces significant upregulation of PIM1, which in turn promotes tumor growth and metastasis by enhancing glycolytic flux. Knockdown of PIM1 in HCC cells leads to reduced glucose uptake and decreased levels of p-AKT and key glycolytic enzymes, further confirming its role in facilitating glycolysis-driven tumor progression [78]. Beyond glucose metabolism, PIM1 also influences amino acid and nucleotide metabolism. In lipid metabolism, PIM1 drives lipid droplet (LD) accumulation, thereby promoting prostate cancer cell proliferation and survival [79]. Mechanistically, PIM1 phosphorylates and inhibits glycogen synthase kinase 3β (GSK3β), leading to the stabilization of peroxisome proliferator-activated receptor alpha (PPARα) and enhanced transcription of genes involved in peroxisome biogenesis factor 3/5 (PEX3/5) and tail-interacting protein of 47 kDa (Tip47), which supports cell proliferation and survival during nutrient deprivation [79]. Additionally, PIM1 can induce peroxisome proliferator-activated receptor γ (PPARγ) expression, thereby promoting fatty acid oxidation (FAO) metabolism and immunosuppressive functions in myeloid-derived suppressor cells (MDSCs) [80]. Collectively, these findings underscore the critical role of PIM1 in metabolic reprogramming and the regulation of the TME. Elucidating the functions and regulatory mechanisms of PIM1 in cancer metabolism not only deepens our understanding of tumor biology but also provides promising new targets and strategies for cancer therapy (Figure 5).

### 3.8. The Role of PIM1 Kinase in Tumor Angiogenesis

Tumor angiogenesis refers to the process by which tumors induce the formation of new blood vessels to secure essential nutrients and oxygen, thereby facilitating their growth and metastatic potential. This process is regulated by a diverse array of cell types and molecular mechanisms, resulting in significant heterogeneity and complexity. Tumor-associated vasculature exhibits marked structural and functional differences compared to normal blood vessels and plays multifaceted roles within the TME, including supporting tumor growth and promoting immune evasion [81]. The abnormal and heterogeneous characteristic of tumor angiogenesis presents major challenges for anti-angiogenic therapies and is a critical factor in tumor progression, invasion, and metastasis [82]. Recent studies have highlighted the pivotal role of PIM1 kinase in regulating tumor angiogenesis. PIM1 directly phosphorylates and stabilizes HIF-1α, thereby promoting angiogenesis in solid tumors through a mechanism that is independent of oxygen concentration [45]. Additionally, PIM1 enhances endothelial cell tube formation and migration by phosphorylating endothelial nitric oxide synthase (eNOS) at Ser-633, further facilitating neovascularization [83]. From a therapeutic perspective, dual inhibition of PIM1 and vascular endothelial growth factor receptor 2 (VEGFR-2) has emerged as a promising strategy to overcome resistance to conventional anti-angiogenic agents, offering synergistic anti-tumor effects [84]. Collectively, these findings indicate that PIM1 kinase contributes to tumor angiogenesis and support its potential as a valuable therapeutic target in cancer treatment.

### 3.9. The Role of PIM1 Kinase in Anti-Cancer Immune Response

**Figure 6:**
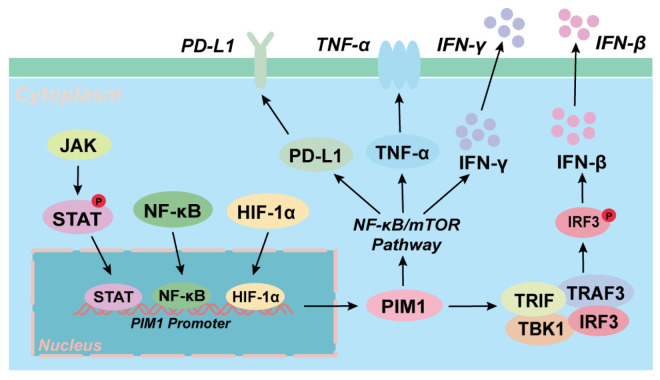
The Role of PIM1 Kinase in Anti-cancer Immune Response. STAT, NF-κB and HIF-1α promote the transcription of PIM1. PIM1 promotes the expression of PD-L1, TNF-α, and IFN-γ via the NF-κB/mTOR pathway. PIM1 enhances IFN-β production by facilitating TRIF/TRAF3/TBK1/IRF3 signaling.

Tumor immunology investigates the interplay between tumor antigens, host immune function, and the processes of tumor initiation, progression, and outcome. Central to this field is the TME, which encompasses all non-cancerous components within the tumor, including metabolites, secreted factors, and a diverse array of immune infiltrating cells such as tumor-infiltrating lymphocytes (TILs) [85]. These immune cells collectively shape the immunological landscape of the tumor, engaging in dynamic interactions with cancer cells and the surrounding stroma. Tumors can modulate their microenvironment by releasing signaling molecules that promote angiogenesis and induce immune tolerance, while immune cells in the TME can influence cancer cell growth [86]. PIM1 plays a critical role in regulating the TME. In prostate cancer, high PIM1 expression is associated with an immunosuppressive TME characterized by elevated inflammation and dense infiltration of suppressive immune cells, most notably tumor-associated macrophages (TAMs) [87]. In NSCLC, PIM1 deficiency promotes TAM reprogramming via inhibition of NF-κB signaling and C-C motif chemokine ligand 2 (CCL2) transactivation, further supporting the potential of PIM1 inhibition as a strategy to enhance cancer immunotherapy [88]. In CRC, PIM1 expression correlates strongly with tumor necrosis factor (TNF)-related immune genes and programmed death-ligand 1 (PD-L1), indicating co-expression with multiple immune checkpoint genes [89]. Beyond its effects on the TME, PIM1 kinases directly regulate immune cell function. PIM1 contributes to innate immune responses by promoting interferon-β (IFN-β) production. Mechanistically, PIM1 enhances the formation of signaling complexes involving TIR-domain-containing adaptor inducing interferon-β (TRIF), TNF receptor-associated factor 3 (TRAF3), TANK-binding kinase 1 (TBK1), and interferon regulatory factor 3 (IRF3), facilitating IRF3 phosphorylation and nuclear translocation, which leads to increased IFN-β synthesis and highlights a novel role for PIM1 in antiviral innate immunity [90]. In summary, PIM1 exerts multifaceted effects in tumor immunology by modulating the TME, regulating immune cell function, and serving as both a therapeutic target and biomarker. These diverse effects highlight the potential of the strategy targeting PIM1 in enhancing cancer immunotherapy and improving clinical outcomes (Figure 6).

### 3.10. The Role of PIM1 Kinase in Therapeutic Resistance

Therapeutic resistance refers to the phenomenon where cancer cells develop resistance to therapeutic agents, posing a significant challenge to effective cancer treatment. PIM1 kinase has emerged as a critical regulator of tumor cell survival, proliferation, and resistance to chemotherapy and radiotherapy in multiple cancers. In NSCLC, PIM1 modulates the TME by regulating the infiltration and polarization of TAMs, thereby enhancing the efficacy of anti-PD-1 therapy. This is primarily mediated through PIM1-driven expression of CCL2, which controls TAM chemotaxis and polarization. Inhibition of PIM1 reduces CCL2 secretion, limiting TAM accumulation and their shift toward a pro-tumor phenotype [88]. PIM1 also contributes to resistance mechanisms in epidermal growth factor receptor (EGFR)-mutant NSCLC by promoting EMT and resistance to osimertinib via the GSK3β signaling pathway [20]. In T-cell lymphomas (TCLs), PIM1 upregulates c-Myc expression, protecting tumor cells from apoptosis and conferring resistance to doxorubicin [23]. In TNBC, PIM1 expression is closely linked to cell proliferation and chemoresistance. Its inhibition reduces the expression of anti-apoptotic factor B-cell lymphoma-2 (BCL2) and prevents mitochondrial-mediated apoptosis, as revealed by dynamic BCL2 homology domain 3 (BH3) profiling [73]. In prostate cancer, PIM1 overexpression is associated with tumorigenesis, castration resistance, and docetaxel resistance [33]. Mechanistically, PIM1 phosphorylates the AR, regulating its degradation and function, thereby promoting the development of castration-resistant prostate cancer under low androgen conditions [91]. Furthermore, PIM1 signaling upregulates MET and other receptor tyrosine kinases (RTKs), contributing to resistance against AKT inhibitors [92]. In PDAC, hypoxia-induced PIM1 overexpression significantly enhances tumor cell resistance to oxaliplatin and 5-fluorouracil under low oxygen conditions [93]. In MET-amplified NSCLC cells, treatment with MET inhibitors leads to upregulation of PIM1, and the use of PIM inhibitors increases the sensitivity of resistant cell lines to MET inhibition [94]. In CRC, miR-3135b directly suppresses PIM1 expression, thereby enhancing sensitivity to 5-fluorouracil [95]. Additionally, inhibition of PIM kinases sensitizes neuroblastoma cells to doxorubicin [96]. Collectively, these findings highlight the pivotal role of PIM1 kinase in mediating therapeutic resistance in diverse cancers through multiple mechanisms that influence tumor cell survival and response to treatment. Targeting PIM1 not only has the potential to enhance the efficacy of existing therapies but also provides a theoretical and practical foundation for the development of novel therapeutic strategies.

## 4. Progress in Drugs Targeting PIM1 Kinase

### 4.1. Summary and Classification of Existing PIM Kinase Inhibitors

PIM kinases have emerged as promising therapeutic targets for personalized treatment of advanced cancers, with several inhibitors developed and evaluated at various stages of clinical trials. These inhibitors are characterized by diverse heterocyclic scaffolds, including pyrroles, pyrimidines, thiazolidines, benzofurans, indoles, triazoles, oxadiazoles, and quinoline derivatives [42]. Notable PIM inhibitors have undergone clinical assessment in cancer patients [97,98,99] (Table 1). However, their efficacy has not fully reached expectations. Recent synthetic efforts have focused on optimizing the potency and selectivity of PIM inhibitors. For example, novel chemical compounds such as GNE-955 were discovered bearing 5-azaindazole core with noncanonical hydrogen bonding to the hinge [100]. Besides, a series of pyrrolo[2,3-a]carbazole derivatives have been tested for inhibitory activity against PIM-1, PIM-2, and PIM-3 kinases, with pyrrolo[2,3-a]carbazole-3-carbaldehyde demonstrating strong PIM1 inhibition and notable antiproliferative effects *in vitro* [101]. Among pyrimidine derivatives, substituted benzofuro[3,2-d]pyrimidinones have shown pan-PIM inhibitory activity, with alkylated benzofuro[3,2-d]pyrimidinone 17 exhibiting potent inhibition. Structural optimization revealed that an 8-bromo substituent is critical for activity [102]. Thiazolidine derivatives, such as (Z)-5-((2-aminopyrimidin-4-yl) methylene) thiazolidine-2,4-dione, have been screened for PIM kinase inhibition, and newly identified compounds like 5-(1H-indol-5-yl)-1,3,4-thiadiazol-2-amine display excellent inhibitory efficacy against all three PIM isoforms [103]. Benzofuran derivatives, including a series of benzofuran-2-carboxylic acids, have been synthesized as PIM-1 inhibitors [104]. Indole derivatives, particularly 3,5-disubstituted indoles, have also demonstrated PIM kinase inhibitory activity [105]. Triazole derivatives, inspired by the casein kinase 2 (CK2) inhibitor CX-4945, have led to the development of novel pan-PIM inhibitors, where the introduction of secondary amide or triazole groups at the C-7 position and halogenated anilines at the C-5 position resulted in compounds with robust activity, typically interacting with the serine 112 residue of PIM kinases, which is crucial for antiproliferative effects [106]. Substituents such as 2-chloro, fluoro, and methyl groups on the C-5 phenyl ring further enhance activity. Quinoline derivatives have shown effective bioactivity against PIM kinases, with antiproliferative potency dependent on the presence of secondary amines and pyridine rings attached to the quinoline core [107]. Pyrazine derivatives, specifically 2,6-disubstituted pyrazines, have been developed as CK2 kinase inhibitors, and structure-guided optimization of 5-substituted-3-thiophene carboxylic acids has yielded lead compounds with inhibitory effects in both enzymatic and cellular assays [108]. Overall, the structural diversity and ongoing optimization of PIM kinase inhibitors underscore their therapeutic potential. Continued research into their molecular mechanisms and clinical efficacy is essential for advancing these compounds as viable options in targeted cancer therapy.

### 4.2. Limitations and Evaluation of Existing PIM1 Inhibitors

#### 4.2.1. Kinase Selectivity and Off-Target Toxicity

One of the primary challenges in the development of PIM1 inhibitors is achieving sufficient kinase selectivity to minimize off-target effects and unpredictable toxicity. The ATP-binding pocket of PIM1 kinases features a unique “proline hinge” structure, which theoretically allows for greater selectivity compared to other kinases. However, early inhibitors such as SGI-1776, despite demonstrating efficacy, exhibited poor selectivity for other kinases like fms-like tyrosine kinase 3 (FLT3) and casein kinase 1δ (CK1δ), resulting in unexpected off-target toxicities [109] (Table 1). There are also many other similar inhibitors that bind to the ATP pocket of PIM1, including INCB053914, Hispidulin, SMI-4a, SC 204330, and PIM1-IN-2 [99,110,111,112,113]. Furthermore, the lack of subtype selectivity among PIM family members (PIM1, PIM2, and PIM3), which share overlapping yet distinct structural and functional characteristics, complicates therapeutic strategies [114]. Pan-PIM inhibitors may offer synergistic anti-tumor effects but also increase toxicity and obscure the specific contributions of each isoform to physiological and pathological processes, thereby hindering precision medicine approaches. For example, PIM2 plays a more prominent role in lymphocyte function and metabolic regulation, and its inhibition may be associated with distinct safety profiles [115] (Table 1). In addition, there are also some bisubstrate inhibitors, like SEL24-B489 that can simultaneously inhibit PIM1 and other molecules [98]. The application of these inhibitors may lead to the activation of some compensatory signaling pathways, thereby weakening the therapeutic effect of the inhibitors. Small-molecule inhibitors that target the distinctive ATP-binding pocket of PIM1 often exhibit poor specificity and considerable off-target toxicity. As a result, research interest has increasingly shifted toward alternative PIM1 kinase inhibitors, in particular substrate-competitive peptides. Synthetic peptides derived from pseudo-substrate sequences have been shown to inhibit PIM1 kinase activity with high selectivity [116]. Recently, a research team developed the peptide inhibitor R8-T198wt, which binds to PIM1 and suppresses PIM1-mediated phosphorylation of endogenous p27^Kip1^ and Bad [117] (Table 1). Studies have demonstrated that the PIM1 kinase inhibitor TP-3654 and genetic ablation of PIM1 prevent the development of myelofibrosis induced by JAK2V617F and MPLW515L, suggesting that TP-3654 may be beneficial for the treatment of myelofibrosis (MF) [118]. Moreover, TP-3654 has received Food and Drug Administration (FDA) Fast Track Designation for the treatment of myelofibrosis. Recently, the first PIM1 PROTAC (PIMTAC) was successfully developed, and the research team demonstrated that PIMTAC degrades PIM1, PIM2, and PIM3 in prostate cancer cell lines, exhibiting superior therapeutic efficacy compared with small-molecule PIM inhibitors both *in vitro* and *in vivo* [119]. This finding also highlights the considerable potential of targeted PIM degradation for cancer therapy.

#### 4.2.2. Resistance and Compensatory Pathways

The efficacy of PIM1 inhibitors as monotherapies is limited by the highly redundant and adaptive nature of cancer signaling networks. Inhibition of PIM1 often triggers compensatory activation of parallel pathways, such as persistent STAT5 activation via the JAK/STAT pathway or upregulation of upstream signals, which can attenuate the anti-cancer effects of PIM1 inhibition [11]. Additionally, activation of the phosphoinositide 3-Kinase (PI3K)/AKT/mTOR pathway, due to overlapping substrate phosphorylation (e.g., Bad and 4E-BP1) between PIM1 and AKT, may relieve negative feedback on AKT signaling, thereby promoting acquired resistance [120]. The therapeutic efficacy of PIM1 inhibitors is also highly dependent on genetic context, such as MYC amplification or FLT3-internal tandem duplication (ITD) mutations, with response rates being significantly higher in biomarker-defined populations (e.g., FLT3-ITD-positive AML or MYC-driven lymphomas) [121]. In the absence of robust biomarkers, monotherapy response rates remain low, limiting broader clinical application.

#### 4.2.3. Pharmacokinetic and Drug-like Properties

Many PIM kinase inhibitors are derived from purine or pyrido[3,4-d]pyrimidinone scaffolds, which often suffer from poor solubility, significant first-pass metabolism, and rapid clearance, resulting in low oral bioavailability and fluctuating plasma concentrations. For instance, AZD1208 demonstrated potent preclinical activity but exhibited a short half-life in phase I clinical trials, necessitating frequent dosing to maintain target coverage [122]. This not only reduces patient compliance but also leads to pronounced peak-trough differences, potentially allowing for target reactivation during trough periods. Moreover, most PIM inhibitors are unable to effectively penetrate the blood–brain barrier due to their molecular polarity and status as P-glycoprotein substrates, limiting their utility in treating primary or metastatic central nervous system (CNS) malignancies [123].

#### 4.2.4. Clinical Development and Biomarker Limitations

The lack of robust pharmacodynamic biomarkers poses a significant challenge for clinical dose optimization and efficacy assessment. While phosphorylation of known PIM1 substrates (e.g., p-Bad Ser112, p-4E-BP1 Ser65) can be measured, it remains uncertain whether changes in these markers in accessible tissues (such as peripheral blood mononuclear cells) reliably reflect PIM1 inhibition in tumor tissue [124,125]. This uncertainty complicates dose selection and pharmacodynamic evaluation in clinical trials. Additionally, early clinical studies often enrolled unselected patient populations without rigorous biomarker stratification, diluting potential efficacy signals in biomarker-enriched cohorts (e.g., PIM1 overexpression, MYC amplification, FLT3-ITD mutation). Rational design of combination therapies is also lacking, with some early trials failing to adequately consider drug–drug interactions, toxicity profiles, and pharmacokinetic compatibility, resulting in additive toxicity and premature trial termination [118]. Despite the promise of PIM1 as an anti-cancer target, the development of its inhibitors highlights several quintessential challenges in modern drug discovery, including selectivity, resistance mechanisms, pharmacokinetic limitations, biomarker development, and rational clinical trial design. Addressing these issues will be critical for realizing the full therapeutic potential of PIM1-targeted therapies.

**Table 1:** Clinical assessment of PIM1 inhibitors.

Drug Name	Chemical	Phase	Application	Inhibitor Category
PIM-447 [97]	Aminocycloexylpyridinecarbamic acid derivative	Clinical trial phaseI	Refractory multiple myeloma	Pan-PIM inhibitor
GDC-0339 [97]	5-Aminothiazole-4-carboxamide	Clinical trial	Multiple myeloma	Pan-PIM inhibitor
TP-3654 [97]	imidazo[1,2-b] pyridazine derivative	Fast Track Designation	Urothelium carcinoma	Pan-PIM inhibitor
SEL24-B489 [98]	Benzimidazole derivative	Preclinical study	Acute myeloid leukaemia	Pan-PIM inhibitor bisubstrate inhibitor
INCB053914 [99]	Cyclopentane[b]pyridine + phenyl-diflorophenyl-fluoropyridinecarboxamide	Clinical trial suspended	/	ATP-competitive inhibitor Pan-PIM inhibitor
GNE-955 [100]	5-azaindazole derivative	Preclinical study suspended	Hematological malignancy	Pan-PIM inhibitor
Hispidulin [110]	Flavone derivative	Preclinical study	/	ATP-competitive inhibiton
SMI-4a [111]	5-(3- Trifluoromethylbenzylidene) thiazolidine-2,4-dione	Preclinical study	Precursor T-cell Lymphoblastic Lymphoma	ATP-competitive inhibiton
SC 204330 [112]	1,2-dihydropyridin-2-one derivative	Preclinical study	/	ATP-competitive inhibiton
PIM1-IN-2 [113]	4-[3-(4-Chlorophenyl)-2,1-benz	Preclinical study	/	ATP-competitive inhibiton
R8-T198wt [117]	p27^Kip1^ Peptide	Preclinical study	prostate cancer	peptidic inhibitor
PIMTAC [119]	PIM1 PROTACS	Preclinical study	/	PROTACS
AZD1208 [122]	Thiazolidinedione derivative	Clinical trial suspended	/	Pan-PIM inhibitor
SGI-1776 [126]	imidazo[1,2-b] pyridazine derivative	Clinical trial suspended	/	ATP-competitive inhibitor Pan-PIM inhibitor
CX-6258 [127]	2-Indoleone derivative	Clinical trial	/	Pan-PIM inhibitor

## 5. Therapeutic Strategies and Challenges in Targeting PIM1

### 5.1. Therapeutic Strategies Targeting PIM1 Kinase in Cancer

A variety of therapeutic strategies have been developed to target PIM1 kinase in cancer, often in combination with other agents to enhance efficacy. In prostate cancer, PIM1-mediated phosphorylation of the AR and its coactivator 14-3-3 ζ coordinates their interaction, suggesting that combined therapy with PIM1 inhibitors and androgen-targeting agents may yield improved clinical outcomes [43]. While monotherapy with PIM1 inhibitors such as NMS-P645 can reverse PIM1-driven pro-survival signaling, such as STAT3 activation via Tyr705 phosphorylation and resistance to taxane-based chemotherapy, it does not elicit robust antitumor effects on its own [128]. However, the combination of NMS-P645 with the PI3K inhibitor GDC-0941 induces a significant antiproliferative response in prostate cancer cells, highlighting the potential of combinatorial approaches [129]. In CRC, HCI-48, a chalcone derivative, demonstrates antitumor activity by selectively inhibiting PIM1 and FGFR1 kinases in an ATP-dependent manner [30]. Chalcones are known for their anti-tumor, anti-inflammatory, and antimicrobial properties, and HCI-48’s dual targeting mechanism provides a promising therapeutic avenue [30]. The functional interplay between STAT3 and PIM1 is a critical signaling event in cancer cell biology. Treatment with curcumin disrupts this interaction and downregulates both STAT3 and PIM1 expression [130,131]. PIM1 is also recognized as a poor prognostic factor in NSCLC. The molecular inhibitor CX-6258 HCl sensitizes NSCLC cells to osimertinib by inhibiting STAT3 phosphorylation, suggesting that co-targeting PIM1 and STAT3 may enhance the therapeutic efficacy of osimertinib [127]. In addition, PIM1 has been shown to mitigate cisplatin-induced acute kidney injury (AKI) by suppressing Dynamin-related protein 1 (Drp1) activation, indicating a potential protective role in chemotherapy-associated toxicity [132]. Recent studies have identified pyrazolo[3,4-b]pyridine derivatives as promising dual cyclin-dependent kinase 2 (CDK2)/PIM1 inhibitors, which effectively suppress both kinases and reduce TNF-α expression, exhibiting potent and selective anticancer activity [133]. Overall, the effective therapeutic strategies often involve the combination of PIM1-targeted inhibitors or PIM1 PROTACs with hormonal agents or other targeted therapies. Immunotherapy is also gaining increasing attention, and the integration of PIM1 inhibitors with immunotherapeutic approaches may represent a highly effective future direction for cancer treatment. These advances underscore the importance of PIM1 as a therapeutic target and highlight the potential of combination regimens to overcome resistance and improve patient outcomes.

### 5.2. Therapeutic Challenges and Future Directions in Targeting PIM1

Despite the potential of PIM1 as a therapeutic target in cancer, several limitations impede the effectiveness of PIM1 inhibitors. Owing to these limitations, no PIM1 inhibitor has been approved for clinical use to date. All candidates have been discontinued during clinical trials. PIM kinases are generally considered weak oncogenes and often require cooperation with other oncogenic pathways to drive tumorigenesis. As a result, single-agent inhibition of PIM1 frequently fails to produce robust anti-tumor responses [134]. Additionally, the clinical application of PIM1 inhibitors is restricted by toxicity, off-target effects at higher doses, and the activation of compensatory signaling pathways, which collectively limit the safe and effective dosing of these agents. Clinical data showed that SGI-1776 exhibited cardiotoxicity and off-target effects on FLT3 and CK1δ. AZD1208 had a short half-life and limited clinical efficacy. Clinical studies have demonstrated that monotherapy with PIM1 inhibitors often yields suboptimal outcomes, and escalating doses may increase the risk of adverse reactions due to off-target activity [135]. The PIM1 inhibitor INCB053914 exhibits limited single-agent activity, and its combination with other agents results in additive toxicity. Beyond these obstacles, therapeutic targeting of PIM1 is further complicated by functional redundancy among PIM family isoforms, which may compromise the effectiveness of selective PIM1 inhibition, and by the absence of validated pharmacodynamic biomarkers for monitoring target engagement and drug response. These challenges highlight the necessity for more advanced therapeutic strategies beyond single-target inhibition. Looking forward, research is increasingly focused on the development of highly selective PIM1 inhibitors, PIM1 PROTACs and the implementation of combination therapies. Structure-based drug design is being utilized to enhance the selectivity and potency of PIM1 inhibitors, thereby reducing off-target toxicity. Concurrently, combining PIM1 inhibitors with other targeted agents or conventional chemotherapeutics is being actively explored to overcome adaptive resistance and improve clinical outcomes. Given the redundancy and compensatory mechanisms within oncogenic signaling networks, multi-targeted approaches, such as dual PIM/PI3K inhibitors, or rationally designed combination regimens may provide superior efficacy by simultaneously disrupting multiple cooperative pathways involved in tumor progression and drug resistance. Although the clinical translation of these strategies remains challenging, such approaches represent a promising direction for future cancer therapy, with the potential to more effectively suppress tumor growth and prevent the emergence of resistance.

## 6. Perspectives on PIM1 Kinase in Tumor Diagnosis and Therapy

PIM1 is overexpressed in a wide range of malignancies, and its unique biological properties make it a promising target for cancer diagnosis and treatment. The advantages of targeting PIM1 are primarily reflected in three aspects. First, PIM1 exerts dual regulatory effects on tumor growth and immune modulation. By phosphorylating downstream targets, PIM1 accelerates cell cycle progression, regulates metabolic and proliferative pathways. Additionally, PIM1 mediates the degradation of interferon alpha and beta receptor subunit 1 (IFNAR1) via the ubiquitin ligase β-transducin repeat-containing protein (β-TrCP), thereby attenuating innate immune responses and potentially facilitating tumor immune evasion [136]. Second, PIM1 is closely associated with therapeutic resistance and tumor recurrence. It plays a central role in the maintenance of CSCs and the development of chemoresistance [20]. Third, PIM1 is involved in a broad regulatory network, participating in multiple signaling pathways such as STAT3, GSK3β, and mTOR, which influence apoptosis, metabolism, immune escape, and DNA damage repair [20,45,68]. This positions PIM1 as a pivotal target for intervention in diverse tumor types. Current strategies for targeting PIM1 can be categorized into single-agent inhibitors, PROTACs and combination therapies three main approaches. Pyridine-triazole derivatives, competitively or non-competitively inhibit PIM1, induce apoptosis via caspase 3/7 activation, and exhibit low toxicity to normal cells [137]. Given the limited efficacy of single-agent inhibitors. The success of PIM1-targeted therapy depends on optimizing selectivity and rational combination strategies. The unique structural features of PIM1, such as the hinge region Pro123, allow for the design of highly selective inhibitors, though off-target effects must be carefully avoided. Recently, a research team developed the first PIM1 PROTAC (PIMTAC), representing a major breakthrough in PIM1-targeted drug development. PROTACs not only inhibit PIM1 activity but also overcome drug resistance arising from mutations in the ATP-binding pocket. Combining PIMTAC with other agents may offer new hope for addressing drug resistance in cancer therapy. As PIM1 promotes DNA damage repair, the combination of PROTACs with DNA synthesis inhibitors may yield promising therapeutic outcomes in tumors with high PIM1 expression. Furthermore, given the role of PIM1 in immune regulation within the TME, combining PIM1 inhibitors with immune checkpoint blockade may represent a future direction. However, there is currently a lack of specific studies on the combination of PIM1 inhibition and immunotherapy. Future research may fill this gap by exploring the therapeutic efficacy of PIM1 inhibitors combined with immune checkpoint blockers. The limitations of current PIM1 inhibitors are multifactorial and systemic, spanning molecular design, preclinical biological understanding, and clinical translation. Future molecular design should leverage structural biology to develop next-generation inhibitors with improved selectivity, metabolic stability, and CNS penetration. Allosteric inhibitors may offer breakthroughs in overcoming selectivity and resistance issues. Rational combination therapies, based on a comprehensive understanding of PIM1 signaling networks, should be developed with downstream effectors (e.g., mTORC1/2) or parallel pathways (e.g., PI3K/AKT), and their synergistic effects and safety profiles must be thoroughly validated in preclinical models. Precision clinical development is essential, employing a precision medicine approach to identify patient populations most likely to benefit from PIM1 inhibition (based on PIM1 expression levels, gene fusions, or mutation status). The integration of reliable pharmacodynamic biomarker analyses into clinical trials will be critical for confirming target inhibition and guiding optimal biological dosing (OBD), rather than relying solely on maximum tolerated dose (MTD). It is recommended that dynamic monitoring of PIM1 activity in circulating tumor cells (CTCs) be developed as a pharmacodynamic biomarker, rather than relying solely on peripheral blood mononuclear cells. In addition, emphasis should be placed on adaptive trial designs, in which combination therapies are adjusted based on real-time biomarkers, thereby yielding more accurate clinical data. In summary, PIM1 represents a multifaceted and promising target in cancer diagnosis and therapy. Continued research into its molecular functions, regulatory mechanisms, and therapeutic targeting strategies will be vital for advancing precision oncology and improving patient outcomes.

## Data Availability

No new data were created or analyzed in this study. Data sharing is not applicable to this article.

## Abbreviations

The following abbreviations are used in this manuscript:

PIM1	Provirus integration site for Moloney murine leukemia virus 1
JAK	Janus Kinase
STAT	Signal Transducer and Activator of Transcription
NF-κB	Nuclear Factor kappa-B
NSCLC	Non-small cell lung cancer
c-MET	Cellular mesenchymal-epithelial transition factor
c-Myc	Cellular myelocytomatosis oncogene
TNBC	Triple-negative breast cancer
ABI2	Abl interactor 2
CRC	Colorectal cancer
IRAK4	Interleukin-1 receptor-associated kinase 4
TLR	Toll-like receptor
MYD88	Myeloid differentiation primary response 88
FGFR1	Fibroblast growth factor receptor 1
ATP	Adenosine triphosphate
PXXP	Proline-X-X-Proline
AR	Androgen receptor
HIF-1α	Hypoxia-inducible factor 1-alpha
PHD	Prolyl hydroxylase domain
PRAS40	Phosphorylates Proline-rich Akt substrate of 40 kDa
AKT	Protein kinase B
mTOR	Mammalian target of rapamycin
4E-BP1	eIF4E-bind-ing protein 1
p70S6K	70 kDa ribosomal Protein S6 kinase
Bad	BCL2-associated agonist of cell death
REDD1	Regulated in development and DNA damage responses 1
AMPK	AMP-activated protein kinase
AAA	Abdominal aortic aneurysm
DLBCL	Diffuse large B-cell lymphoma
BCR	B-cell receptor
DDR	DNA damage response
HR	Homologous recombination
NHEJ	Non-homologous end joining
DSBs	DNA double-strand breaks
DNA-PKcs	DNA-dependent protein kinase catalytic subunit
PDLIM1	PDZ And LIM Domain 1
ATM	Ataxia telangiectasia mutated
CHK2	Checkpoint kinase 2
MDC1	Mediator of DNA damage checkpoint protein
53BP1	p53 binding protein 1
PARP	Poly ADP-ribose polymerase
SASP	Senescence-associated secretory phenotype
OIS	Oncogene-induced senescence
SND1	Staphylococcal nuclease domain-containing protein 1
HP1-γ	Heterochromatin protein 1γ
H3K9me3	Histone H3 lysine 9 trimethylation
UHRF1	Ubiquitin-like with PHD and ring finger domains 1
p16	Cyclin-dependent kinase inhibitor 2A
IL-6	Interleukin-6
CBX8	Chromobox homolog 8
PML	Promyelocytic leukemia protein
p27	Cyclin-dependent kinase inhibitor 1B
HCC	Hepatocellular carcinoma
ROCK2	Rho-associated coiled-coil-containing protein kinase 2
RBMY	RNA-binding motif protein, Y chromosome
ROS	Reactive Oxygen Species
NDRG1	N-myc downstream regulated 1
NFATC1	Nuclear factor of activated T cells 1
PDAC	Pancreatic ductal adenocarcinoma
SMAD	SMAD family member
K-Ras	Kirsten rat sarcoma viral oncogene homolog
BCL6	B-cell lymphoma 6
RUNX3	RUNX family transcription factor 3
CD133	Prominin-1
CSCs	Cancer stem cells
NuMA1	Nuclear mitotic apparatus protein 1
ALDH	Acetaldehyde dehydrogenase
BCRP	Breast cancer resistance protein
LD	Lipid droplet
GSK3β	Glycogen synthase kinase 3β
PPARα	Peroxisome proliferator-activated receptor alpha
PEX3/5	Peroxisome biogenesis factor 3/5
Tip47	Tail-interacting protein of 47 kDa
PPARγ	Peroxisome proliferator-activated receptor γ
FAO	Fatty acid oxidation
MDSCs	Myeloid-derived suppressor cells
eNOS	Endothelial nitric oxide synthase
VEGFR-2	Vascular endothelial growth factor receptor 2
TME	Tumor microenvironment
TILs	Tumor-infiltrating lymphocytes
TAMs	Tumor-associated macrophages
TIL	Tumor-infiltrating lymphocytes
TAM	Tumor-associated macrophages
CCL2	C-C motif chemokine ligand 2
TNF	Tumor necrosis factor
PD-L1	Programmed death-ligand 1
IFN-β	Interferon-β
TRIF	TIR-domain-containing adaptor inducing Interferon-β
TRAF3	TNF receptor-associated factor 3
TBK1	TANK binding kinase 1
IRF3	Interferon regulatory factor 3
EGFR	Epidermal Growth Factor Receptor
TCLs	T-cell lymphomas
BCL2	B-cell lymphoma-2
RTKs	Receptor tyrosine kinases
CK2	Casein kinase 2
FLT3	Fms-like tyrosine kinase 3
CK1δ	Casein Kinase 1δ
MF	Myelofibrosis
FDA	Food and Drug Administration
PIMTAC	PIM1 PROTAC
PI3K	Phosphoinositide 3-Kinase
ITD	Internal Tandem Duplication
CNS	Central nervous system
AKI	Acute kidney injury
Drp1	Dynamin-related protein 1
CDK2	Cyclin-dependent kinase 2
IFNAR1	Interferon alpha and beta receptor subunit 1
β-TrCP	β-transducin repeat-containing protein
OBD	Optimal biological dosing
MTD	Maximum tolerated dose
CTCs	Circulating tumor cells
